# *De novo* whole-genome assembly of a wild type yeast isolate using nanopore sequencing

**DOI:** 10.12688/f1000research.11146.2

**Published:** 2018-08-03

**Authors:** Michael Liem, Hans J. Jansen, Ron P. Dirks, Christiaan V. Henkel, G. Paul H. van Heusden, Richard J.L.F. Lemmers, Trifa Omer, Shuai Shao, Peter J. Punt, Herman P. Spaink

**Affiliations:** 1 Institute of Biology, Leiden University, Leiden, 2300 RA, The Netherlands; 2 Future Genomics Technologies B.V., Leiden, 2333 BE, The Netherlands; 3 Department of Human Genetics, Leiden University Medical Center, Leiden, 2333 ZA, The Netherlands; 4 Dutch DNA Biotech B.V., Utrecht, 3584 CH, The Netherlands

**Keywords:** Nanopore sequencing, de novo genome assembly, wild type yeasts, ethanologenic, Candida, Cyberlindera

## Abstract

**Background** *:* The introduction of the MinION sequencing device by Oxford Nanopore Technologies may greatly accelerate whole genome sequencing. Nanopore sequence data offers great potential for *de novo* assembly of complex genomes without using other technologies. Furthermore, Nanopore data combined with other sequencing technologies is highly useful for accurate annotation of all genes in the genome. In this manuscript we used nanopore sequencing as a tool to classify yeast strains.

**Methods** *:* We compared various technical and software developments for the nanopore sequencing protocol, showing that the R9 chemistry is, as predicted, higher in quality than R7.3 chemistry. The R9 chemistry is an essential improvement for assembly of the extremely AT-rich mitochondrial genome. We double corrected assemblies from four different assemblers with PILON and assessed sequence correctness before and after PILON correction with a set of 290 Fungi genes using BUSCO.

**Results** *:* In this study, we used this new technology to sequence and *de novo* assemble the genome of a recently isolated ethanologenic yeast strain, and compared the results with those obtained by classical Illumina short read sequencing. This strain was originally named *Candida vartiovaarae* ( *Torulopsis vartiovaarae*) based on ribosomal RNA sequencing. We show that the assembly using nanopore data is much more contiguous than the assembly using short read data. We also compared various technical and software developments for the nanopore sequencing protocol, showing that nanopore-derived assemblies provide the highest contiguity.

**Conclusions** *:* The mitochondrial and chromosomal genome sequences showed that our strain is clearly distinct from other yeast taxons and most closely related to published *Cyberlindnera* species. In conclusion, MinION-mediated long read sequencing can be used for high quality *de novo *assembly of new eukaryotic microbial genomes.

## Introduction

With the development of robust second generation bioethanol processes, next to the use of highly engineered *Saccharomyces cerevisiae* strains ^1, 2^, non-classical ethanologenic yeasts are also being considered as production organisms ^3, 4^. In particular, aspects concerning the ability to use both C6 and C5 C-sources and feedstock derived inhibitor resistance have been identified as important for the industrial applicability of different production hosts ^3^. In our previous studies we have identified a novel ethanologenic yeast, *Wickerhamomyces anomala*, as a potential candidate ^3^. Based on this research, a further screen for alternative yeast species was initiated (Punt and Omer, unpublished study) Here we describe the isolation and genomic characterization of one of these new isolates, which was typed as *Candida vartiovaarae* based on ribosomal RNA analysis.

With the arrival of next generation sequencing and the assemblers that can use this type of sequencing data, whole genome shotgun sequencing of completely novel organisms has become affordable and accessible. As a result, a wealth of genomic information has become available to the scientific community leading to many important discoveries. While generating whole draft genomes has become accessible, these genomes are often fragmented due to the nature of these short read technologies ^5^. Assembling short read data into large contigs proved to be difficult because the short reads do not contain the information to span repeated structures in the genome. Approaches to sequence the ends of larger fragments partially mitigated this problem ^6^.

The new long read platforms from Pacific Biosciences and Oxford Nanopore Technologies made it possible to obtain reads that span many kilobases ^7^. Assemblies using this type of data are often more contiguous than assemblies based on short read data ^8, 9^.

We have employed the Oxford Nanopore Technologies MinION device to sequence genomic DNA from the isolated *Candida vartiovaarae* strain. The same DNA was also used to prepare a paired end library for sequencing on the Illumina HiSeq2500. The sequence data were used in various assemblers to obtain the best assemblies.

## Materials and methods

### Strain selection and cultivation conditions

In our previous research ^3^, a screening approach was developed to select for potential ethanologens using selective growth on industrial feedstock hydrolysates. Based on this approach, a previously identified microflora from grass silage was screened for growth on different hydrolysates from both woody and cereal residues. From this microflora, a strain was isolated (DDNA#1) after selection on a growth medium consisting of 10% acid-pretreated corn stover hydrolysate, which was shown to be most restrictive in growth due to the presence of relatively high amounts of furanic inhibitors.

### DNA purification

Cells were grown at 30°C on plates with YNB (without amino acids) medium supplemented with 0.5% glucose. Cells were scraped from plates and resuspended in 5 ml TE. High MW chromosomal DNA was isolated from yeast isolate DDNA#1 and *Saccharomyces cerevisiae* S288C using a Qiagen Genomic-tip 100/G column, according to the manufacturer’s instructions.

### Pulsed field gel electrophoresis

In order to determine the size of intact chromosomes of DDNA#1, a BioRad CHEF Genomic DNA Plug Kit was used. Briefly, yeast cells were treated with lyticase and the resulting spheroplasts were embedded in low melting point agarose. After incubation with RNase A and Proteinase K, the agarose plugs were thoroughly washed in TE. The DNA in the agarose plugs was separated on a 0.88% agarose gel in 1xTAE buffer on a Bio-Rad CHEF DRII system. The DNA was separated in four subsequent 12 hour runs at 3V/cm; run one and two used a constant switching time of 500 seconds, and in run three and four the switching time increased from 60 seconds to 120 seconds. The gel was afterwards stained with ethidium bromide and imaged.

### Genome size estimation and heterozygosity

A k-mer count analysis was done using Jellyfish ^10^ v2.2.6 on the Illumina data. From the paired end reads, only the first read was truncated to 100 bp to avoid the lower quality part of the read. The second read was omitted from this analysis to avoid counting overlapping k-mers. Different k-mer sizes were used ranging from k=17 to 23. After converting the k-mer counts into a histogram format, this file was analyzed using the Genomescope ^11^ tool, available at http://qb.cshl.edu/genomescope/ and https://github.com/schatzlab/genomescope.

### Illumina library preparation, sequencing and quality control

High molecular weight DNA from both DDNA#1 and *Saccharomyces cerevisiae* S288C was sheared using a nebulizer (Life Technologies). The sheared DNA was used to make genomic DNA libraries using the Truseq DNA sample preparation kit, according to the manufacturer’s instructions (Illumina Inc.). In the size selection step, a band of 330–350 bp was cut out of the gel to obtain an insert length of ~270 bp. From the resulting libraries, 4.5 million fragments were sequenced in paired end reads with a read length of 150 nt on an Illumina HiSeq2500, according to the manufacturer’s instructions. The HiSeq control software (HCS) and real time analysis (RTA) software, versions were 2.2.38 and 1.18.61, respectively, were used. To ensure data integrity we have visualized read quality distributions with FastQC ^12^ v0.11.7 and merged overlapping paired end reads, including trimming of low quality regions, using flash ^13^ v1.2.11. Only trimmed and merged reads are used as input data for both Spades ^14^ assemblies and assembly polishing.

### MinION library preparation, sequencing and quality control

The genomic DNA was sequenced using nanopore sequencing technology. First the DNA was sequenced on R7.3 flow cells. Subsequently, multiple R9 and R9.4 flow cells were used to sequence the DNA. For R7.3 sequencing runs, we prepared the library using the SQK-MAP006 kit from Oxford Nanopore Technologies. In short, high molecular weight DNA was sheared with a g-TUBE (Covaris) to an average fragment length of 20 kbp. The sheared DNA was repaired using the FFPE Repair Mix, according to the manufacturer’s instructions (New England Biolabs). After cleaning the DNA with bead extraction, using a ratio of 0.4:1 Ampure XP beads (Beckman Coulter) to DNA, the DNA ends were polished and an A overhang was added with the NEBNext End Prep Module (New England Biolabs). Then, prior to ligation, the DNA was again cleaned by extraction using a ratio of 1:1 Ampure XP beads to DNA. The adaptor and hairpin adapter were ligated using Blunt/TA Ligase Master Mix (New England Biolabs). The final library was prepared by cleaning the ligation mix using MyOne C1 beads (Invitrogen).

To prepare 2D libraries for R9 sequencing runs, we used the SQK-NSK007 kit from Oxford Nanopore Technologies. The procedure to prepare a library with this kit is largely the same as with the SQK-MAP006 kit. 1D library preparation was done with the SQK-RAD001 kit from Oxford Nanopore Technologies, which tags high molecular weight DNA using a transposase. The final library was prepared by ligation of the sequencing adapters to the tagmented fragments using the Blunt/TA Ligase Master Mix (New England Biolabs).

The prepared libraries were loaded on the MinION flow cell, which was docked on the MinION device. The MinKNOW software (v0.50.2.15 for SQK-MAP006 libraries and v1.0.5 for SQK-NSK007 and SQK-RAD001 libraries) was used to control the sequencing process and the read files were uploaded to the cloud based Metrichor EPI2ME platform for base calling. Base called reads were downloaded in fastq format. We filtered the data to a per read average maximum error-rate distribution of 10% and a minimum of 10 kbp for quality and length, respectively. Only reads that meet these filtering thresholds were used for assemblies and post-assembly error correction.

### Genome assembly and assembly correction

The sequence data from the Illumina platform was assembled using Spades v3.6.0, we performed a two-branch assembly strategy using either exclusively Illumina data or a hybrid approach combining both Illumina and nanopore data sets.

A set of four different assemblers is used to generate contigs exclusively based on nanopore data, Canu ^15^ v1.3, Miniasm ^16^ v0.2, TULIP ^17^ v0.4 and Smartdenovo ^18^ v1.07. These assemblers perform all vs. all alignments on filtered nanopore data to generate the final contigs, with the exception of TULIP, which aligns reads to a set of random 1,000 bp seed sequences comprising 0.5 times the estimated ~12 Mbp genome size. Contigs of all assemblers were post-assembly corrected using Racon ^19^, excluding Canu generated contigs, since Canu contains an integrated self-correction procedure prior to assembly. To obtain optimum sequence correctness the resulting contigs of these four assemblers were polished with Illumina data using PILON ^20^ v1.18 in a double iterative fashion.

The sequencing data, including the final assembly, has been submitted to the European Nucleotide Archive and can be accessed at http://www.ebi.ac.uk/ena/data/view/PRJEB19912.

### Genome assembly assessment based on gene prediction

As successful sequence polishing plausibly improves the accuracy of gene prediction, we assessed both assembly quality and PILON correction effects using BUSCO ^21^ v3.0.2. We assessed our nanopore exclusive assemblies both before and after PILON correction using lineage database Fungi 0db9 containing 290 genes. BUSCO genome assembly assessments on Spades contigs correspond to assessments after PILON correction for nanopore derived contigs, since Spades contigs are based on Illumina data and do not require a post-assembly PILON correction. BUSCO identifies genes in genomic assemblies either as partial, single or double copy, or completely absent.

### Full genome comparison

From 26S ribosomal RNA sequences available in the nucleotide database, Chen *et al*. ^22^ have constructed a phylogenetic tree. From that phylogenetic tree we have observed that the closest relative for which whole genome sequences are available is *Cyberlindnera jadinii*. To compare our draft genome assembly to this yeast species, we retrieved assemblies of two *Cyberlindnera jadinii* strains, namely NBRC 0988 (GenBank accession number, DG000077.1) and CBS1600 (GenBank accession number, CDQK00000000.1). We also used *Saccharomyce cerevisiae* S288C (GenBank accession number, GCA_000146045.2) in this comparison. We aligned those assemblies to the corrected draft assembly of our strain using MUMmer’s alignment generator NUCmer ^23^ v3.1). NUCmer’s output was filtered and the filtered results parsed to MUMmerplot, generating full-genome visualization between the pairs of different yeast species. Since Spades assembly-lengths are roughly twice the estimated genome size we additionally evaluated alignments between Spades hybrid and TULIP contigs. Alignments were performed using BWA-mem ^24^ v0.7.15 with -x ontd2 settings and visualized using genome viewer Tablet ^25^ v1.17.08.17.

### Read mapping to mitochondrial genome

Reads generated on the Illumina platform were aligned to the published *Candida vartiovaarae* mitochondrial genome (Genbank accession number, KC993190.1) using Bowtie2 ^26^ v2.2.5. Reads generated on the MinION platform were aligned using Minimap2 ^27^ v2.3-r546-dirty. Resulting bam files were sorted and viewed in IGV viewer v2.3.

## Results and discussion

### Pure cultures of candidate ethanologenic yeasts

From a screen on 10% acid-pretreated corn stover hydrolysate, about 70 individual clones were obtained, only five of which were able to grow well on purely synthetic YNB-based medium. To determine the taxonomic status of these clones, chromosomal DNA was isolated and used for PCR amplification of the ribosomal ITS sequence using ITS-specific primers ^28^ (ITS1 and ITS4).

BLAST analysis of these ITS sequences of all 5 isolates revealed a 100% identity to *Candida vartiovaarae* ( *Torulopsis vartiovaarae*: NCBI accession number KY102493)

All five isolates were grown on different C-sources and showed growth on glucose, mannose, cellobiose, xylose and glycerol, while growth on L-arabinose was variable. No significant growth was found on galactose and rhamnose. Good growth (on glucose) occurred between 20–30°C, at pH3-7 (optimum 25°C, pH4-5). Based on the results, we concluded that all five isolates originated from a single source in the grass silage sample. Subsequent experiments were therefore carried out with a single isolate now named DDNA#1.

### Pulsed field gel electrophoresis

**Figure 1.**
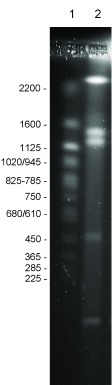
Pulsed field gel electrophoresis of *Candida vartiovaarae* DDNA#1 chromosomes. In lane 1, the chromosomes of *Saccharomyces cerevisiae* were loaded as a marker. Sizes of the chromosomes in the marker lane are indicated. In lane 2, the chromosomes of *Candida vartiovaarae* DDNA#1 were loaded.

As a further means to validate our assembled contigs and determine if they match the actual chromosome length, we have separated the chromosomes on an agarose gel using pulsed field gel electrophoresis. The gel image in Figure 1 shows five bands that represent the chromosomes of this yeast strain. The smallest band has a length that corresponds to the length of the mitochondrial genome (33 kbp). Additional fragments of 450, 1200, and 1500 kbp are also found. The intensity of the band that runs above the 2200 kbp marker band suggests that it actually contains more than one distinct fragment. To make the genome size fit to the estimate derived from the assembly and k-mer analysis (~12.5 Mbp), three ~3 Mbp chromosomes should be postulated. The uncertainty in chromosome size estimate based on pulsed field electrophoresis gels is high because of the large chromosome size and the fact that it is difficult to determine if more than one fragment is present in the gel at a given position. Our conclusion that the top band represents three or more chromosomes is in agreement with the genome sequences of two related *C. jadinii* strains, namely CBS1600 and NBRC 0988.

### Genome size estimation and heterozygosity

**Figure 2.**
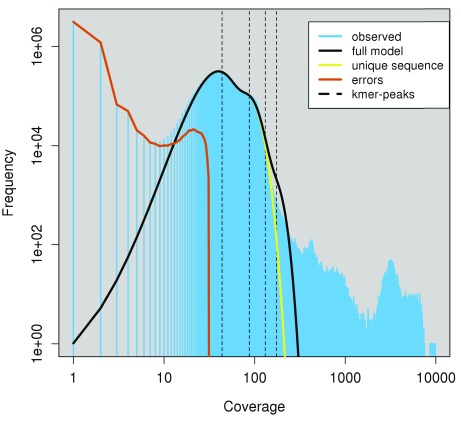
Genome size estimation generated by Genomescope, providing a k-mer analysis (k = 19, from Jellyfish) to estimate haploid genome size, fraction of heterozygosity and coverage. Genomescope attempts to find k-mer count peaks, low and high coverage peaks indicating hetero- and homozygosity. ( **A**) We find ~13× and ~28× coverage for hetero- and homozygous fractions in our dataset. Exact peak positions are determined with a log transformation. Evaluating the slope between coverage points reveals the peak positions indicating hetero- and homozygosity, for lower and higher coverage, respectively.

The Illumina sequence data of our DDNA#1 isolate were submitted to the Genomescope software package to analyze the k-mer count distribution, using k-mer size = 19 at an average coverage of 28.0x ( Figure 2). The ‘haploid’ genome is predicted to contribute to the most abundant fraction, which corresponds with the second peak (dotted line) in the plot ( Figure 2). The first peak corresponds to sequence occurring exactly half as frequently as the main peak, so these are plausibly haplotypes. Due to the nature of k-mer counting, this peak often appears higher than the main peak, because a single SNP will affect all k-mers overlapping that position. The first two peaks contain about 10 Mbp of sequence. Additional peaks at higher coverage indicate duplications and repetitive DNA that are quite abundant, but correspond with less sequence than the second peak. Genomescope estimated a haploid genome size of between 12.00 and 12.01 Mbp. Additionally, Genomescope revealed 3.6% variety across the entire genome indicating that the genome of *C. vartiovaarae* has strong heterozygous properties ( Table 1). A likely possibility is that areas in the genome are replicated and slightly diverged in sequence. This could also explain why we see a large tail of repeated k-mers ( Figure 2). It could also explain why our assembly still remained fragmented despite the relatively large amount of nanopore data that was used in the assembly.

**Table 1.** Most important metrics from Genomescope.

k = 19	k-mer coverage	28.0
property	min	max
Heterozygosity (%)	3.64	3.65
Genome Haploid Length (bp)	11,995,570	12,010,675
Genome Repeat Length (bp)	2,179,917	2,182,662
Genome Unique Length (bp)	9,815,653	9,828,014
Model Fit (%)	98.26	98.89
Read Error Rate (%)	0.13	0.13

### Illumina and MinION *de novo* genome assembly

We took six approaches to assemble the genome of DDNA#1, five assemblies based on sequencing data from a single platform (either Illumina or nanopore) and one hybrid assembly. The first approach used reads exclusively produced by the Illumina platform. After merging paired end reads we obtained ~1.7 Gbp of ~240 bp reads. Contigs generated by Spades remained short and the overall assembly was heavily fragmented. The N50 of this assembly was only ~4.3 kbp, its longest contig ~35 kbp. Spades generated 10,121 contigs and the entire assembly length was nearly twice the estimated ~12 Mbp haploid genome size. We also assembled *Saccharomyces cerevisiae* S288C using a similar short read dataset that was made and sequenced in parallel. Here we obtained an assembly that consisted of 768 contigs with a longer N50 of 124 kbp.

Assembly comparison of *Saccharomyces cerevisiae* and DDNA#1 exclusively based on Illumina data highlights that Spades clearly struggles to reconstruct the genome of our isolate, possibly due to complex SNP arrangements. From these results we take that, even under high coverage conditions, ~240 bp reads do not provide sufficient power to resolve complex SNP distributions for highly heterozygous genomes. This illustrates the necessity of increased read length to fully reconstruct complex genomic structures such as those found in DDNA#1.

Secondly, we used Spades to generate a hybrid assembly that takes both Illumina and nanopore data as input. We used ~1.7 Gbp and ~208 Mbp Illumina and nanopore data sets, respectively. This hybrid approach performed by Spades resulted in an N50 of ~379 kbp, with the longest contig ~1.1 Mbp, and a total of 653 contigs and, although still relatively fragmented, it is interesting that it yielded a similar assembly length compared to the assembly exclusively based on Illumina data. The improvement of assembly statistics strongly indicates the positive effect of longer reads in resolving complicated genomes.

Hereafter, the four remaining approaches are all based on data solely generated by the Oxford Nanopore Technologies platform. Assembly lengths in particular are fairly similar between all four assemblies and all approximate the estimated ~12 Mbp haploid genome size. However, Miniasm, TULIP and Smartdenovo outperform Canu on N50, number of contigs and longest contig ( Table 2). Lengths of the longest contig from both Smartdenovo and TULIP (~2,8 Mbp) corresponds to the suggestion of ~3 Mbp chromosomes shown using pulse field gel electrophoresis on intact chromosomal DNA ( Figure 1). This suggests that both Smartdenovo and TULIP were able to fully reconstruct one of the three largest chromosomes of our isolate. Although Smartdenovo results the lowest number of contigs, which is mainly due to a filtering step that filters out very short contigs (shortest contig lengths 1,716 bp and 73,332 bp for TULIP and Smartdenovo, respectively), TULIP generates the highest contiguity with N25 and N50 both around 1.6 Mbp compared to Smartdenovo that results in 1.4 Mbp and 900 kbp, respectively. Hence based on contiguity we prefer to take the TULIP result as the final assembly.

**Table 2.** Data characteristics and assembly statistics.

Assemblers	Canu	Miniasm	TULIP	Smartdenovo	Spades hybrid	Spades
**Data type**	ONT	ONT	ONT	ONT	ONT and Illumina	Illumina
**Reads (#)**	11,344	11,344	11,344	11,344	11,344	8,628,787
**Coverage (x)**	17	17	17	17	17	135
**GC-cont (%)**	46	46	46	46	46	47
**Bases (#)**	208,357,153	208,357,153	208,357,153	208,357,153	208,357,153	1,688,824,952
**Contigs**	34	25	28	20	653	10.121
**Assembly length (bp)**	11,968,989	12,072,133	11,325,084	11,732,656	22,772,746	22,356,011
**Genome size (Mbp)**	12.5	12.5	12.5	12.5	12.5	12.5
**N25 (bp)**	959,647	1,361,451	1,591,600	1,429,838	824,043	7,876
**N50 (bp)**	805,206	1,020,131	1,586,208	902,730	379,588	4,318
**N75 (bp)**	456,000	506,710	619,623	456,270	200,675	2,041
**Max length (bp)**	1,430,409	1,569,347	2,792,203	2,800,024	1,101,756	34,707
**Mean length (bp)**	352,029	482,885	404,467	586,632	34,874	2,208
**Min length (bp)**	4,727	8,316	1,716	73,332	128	128

It is clear from these results that assemblies based on exclusively nanopore data achieve the most contiguous assemblies, as has been shown previously ^8, 9^.

**Figure 3.**
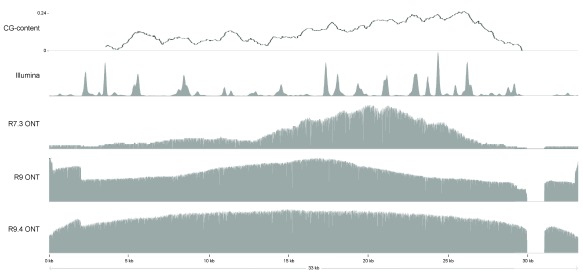
Coverage plot of the *Candida vartiovaarae* DDNA#1 mitochondrial genome. Reads from both the Illumina, and the nanopore platform were aligned to the *Candida vartiovaarae* mitochondrial genome (Genbank accession number, KC993190.1) to show the difference in coverage between the different platforms and chemistry versions.

We also used the nanopore datasets made with the R7.3 and R9 chemistry separately in the Canu assembler. The most notable difference between these assemblies is found in the mitochondrial genome. Only 16 kbp of this 33 kbp genome could be assembled with the R7.3 data, whereas the R9 assembly contained a complete mitochondrial genome (Genbank accession number, KC993190.1). The mitochondrial genome has a very low GC content (21%) and in the extragenic regions more A and T homopolymers are found. Very few R7.3 reads mapped to this region, but in the R9 dataset there are many more reads that represent this region ( Figure 3). It has been shown that the R7.3 data especially has a bias against A and T homopolymers. Although this bias is still not fully absent ^29, 30^, it is reduced for R9 chemestry, indicating technical enhancement and suggesting improved genomic reconstruction even for low complexity regions,. Both after long read self-correction using Canu as well as for post-asssembly correction using Racon the contig sequences still contain errors ^15^. We have used PILON and the complementary Illumina data from this strain to correct the assembled contigs twice. Homopolymer streches are paricularly difficult to base call accurately due to low complexity and lengths are usually underestimated. PILON correction leads to a minor assembly length increase since corrected homopolymer lengths adds to the final assembly size.

### Genome assembly assessment based on gene prediction

**Figure 4.**
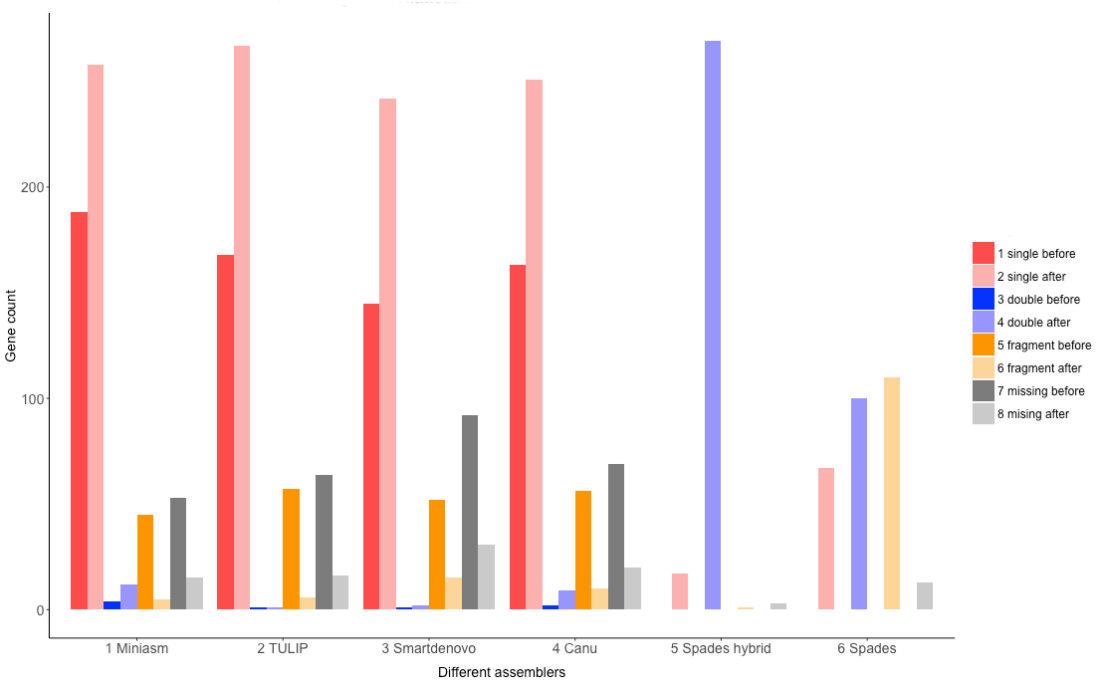
BUSCO genomic assembly assessment using Fungi 0db9 database. Shown on the X-axis are 5 different assembler used in this study, including a hybrid assembly approach performed by Spades. Shown on the Y-axis are the Fungi 0db9 gene counts identified by BUSCO. Dark and light coloring shades indicate before and after PILON correction per classification type, respectively.

BUSCO identifies the majority of genes from database Fungi 0db9 on nanopore derived assemblies. The number of single copy genes identified ranges from 145 to 188, between 45 and 57 genes are partially recognized, and 53 to 92 genes are classified absent before PILON correction ( Figure 4). After PILON correction nearly all genes are identified as single copies in the results from all four assemblers, giving support for the suggestion (based on genome size) that these assemblers yielded haploid genomes. Interestingly, gene identification on Spades contigs, particularly for our hybrid assembly, identified 269 genes as double copy genes. Together with assembly lengths of twice the estimated genome size these results strongly suggest that Spades was able to separately assemble both haplotypes forming a diploid genome under hybrid conditions. Only 100 and 67 genes are identified as double and single copy genes, respectively, for the Illumina exclusive assembly, again indicating the necessity of long read data to maximally reconstruct highly heterozygous genomes.

### Genome comparison

**Figure 5.**
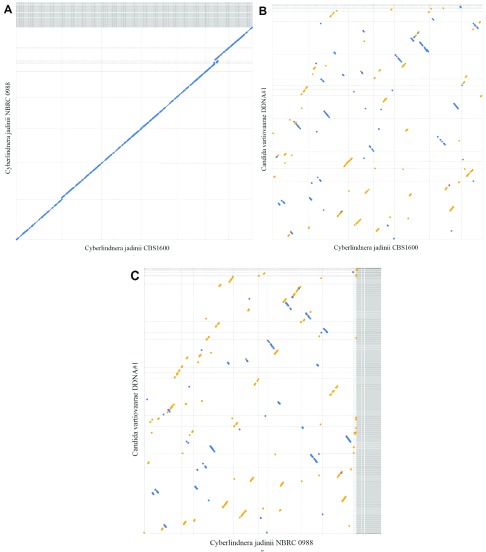
Full genome comparisons between different yeast species. Dashed lines indicate contigs (start and stop positions) and the area between dashed lines indicates the contig size. Blue and yellow dots are hits in reverse and forward orientation, respectively. Diagonal lines indicate sequence and synteny conservation across species. ( **A**) Comparison between NBRC 0988 (vertical axis) and Cyberlindnera jadinii strains CBS1600 (horizontal axis) with 8 kbp as minimal hot length. ( **B**) Comparison between Candida vartiovaarae isolate DDNA#1 (vertical axis) and Cyberlindnera jadinii strain CBS1600 (horizontal axis) with 100 bp as minimal hit length. ( **C**) Comparison between Candida vartiovaarae isolate DDNA#1 (vertical axis) and Cyberlindnera jadinii strain NBRC 0988 (horizontal axis) with 100 bp as minimal hit length.

**Figure 6.**
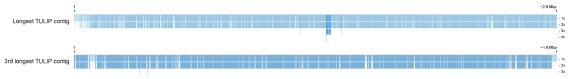
Tablet visualization of Spades hybrid contigs aligned to TULIP contigs. The Spades hybrid contigs aligned against longest TULIP contig (~2.8 Mbp) and the third longest TULIP contig (~1.6 Mbp). White horizontal lines indicate coverage boundaries and show that most regions on the TULIP contigs are covered twice. Alignment gaps come from heavily fragmented Spades hybrid contigs that are aligned on contiguous TULIP contigs. Visualization is based on coverage overview settings in Tablet.

We have compared the assembled contigs of our *C. vartiovaarae* isolate DDNA#1 strain to yeast genome sequences that are already deposited in the nucleotide database. Comparison of our yeast strain with the well characterized *S. cerevisiae* assembly showed negligible genomic similarity. From 26S ribosomal RNA sequences available in the nucleotide database, Chen *et al*. ^22^ have constructed a phylogenetic tree. The closest relatives for which whole genome sequences are available are *C. jadinii* strains CBS1600 and NBRC 0988. An initial comparison between CBS1600 and NBRC 0988 revealed that these two strains show high homology ( Figure 5A). The genomic similarity between our strain and *C. jadinii* strains CBS1600 and NBRC 0988 is much lower ( Figure 5B and Figure 5C, respectively). Assemblies exclusively based on nanopore data compared to Spades hybrid assembly strongly suggests the diploid properties of our strain, at least to a partial extend. At nearly every position on >90% of the TULIP assembly length a Spades hybrid contig is aligned. Figure 6 shows the longest TULIP contig and the third longest TULIP contig, ~2.9 and ~1.6 Mbp, respectively, and alignment of all possible Spades hybrid contigs. For TULIP contigs sorted on length we observe this double coverage behavior for contigs down to ~84 kbp. Shorter TULIP contigs tend to be less consistently double covered or even lack coverage of a Spades hybrid contig all together. In conclusion, these data show that wild type yeast strains are very heterogeneous, despite a high similarity based on ribosomal RNA ITS sequences. Therefore, the data suggest that nanopore sequencing is an essential new tool to classify yeast strains.
