# Supplementary material for: De novo whole-genome assembly of a wild type yeast isolate using nanopore sequencing
Source: F1000Res. 2018 Aug 3;6:618. Originally published 2017 May 3. [Version 2] doi: 10.12688/f1000research.11146.2 (PMC6081980; doi:10.12688/f1000research.11146.2)
Supplement: Peer review history

# Peer review: Referee response for version 2

**DOI:** 10.5256/f1000research.16938.r36802

**Published:** 2018-10-25

**Authors:** Jean-Marc Aury, Istace Benjamin

**Affiliations:** 1 Genoscope, Institut de biologie François-Jacob, Commissariat à l’Energie Atomique (CEA), Université Paris-Saclay, Evry, F-91057, France

The authors submitted a revised version of their manuscript and took into account my main concerns. However, I still have some minor issues to share.

 1) The Tulip assembler require a set of seeds as input, but the authors did not mentioned how those seeds were obtained.

 2) Table 2 refers to a dataset of 11,344 nanopore reads (representing 17X). Does it represent the entire dataset (R7.3 and R9 runs)? In the first version, the authors reported a dataset of 2.05Gb (representing a higher coverage, ~200X). Please clarify this issue by, for example, adding a table which describes the produced dataset and the input dataset used by each assembler.

 3) The filter parameters of the NUCmer output are not mentioned.

We have read this submission. We believe that we have an appropriate level of expertise to confirm that it is of an acceptable scientific standard, however we have significant reservations, as outlined above.

# Peer review: Referee response for version 2

**DOI:** 10.5256/f1000research.16938.r36799

**Published:** 2018-08-24

**Authors:** Mile Šikić

**Affiliations:** 1 Faculty of Electrical Engineering and Computing, University of Zagreb, Zagreb, Croatia

The authors have addressed my concerns sufficiently to recommend indexing of the manuscript in its current form.

I have read this submission. I believe that I have an appropriate level of expertise to confirm that it is of an acceptable scientific standard.

# Peer review: Referee response for version 2

**DOI:** 10.5256/f1000research.16938.r36800

**Published:** 2018-08-07

**Authors:** Christina A. Cuomo

**Affiliations:** 1 Infectious Disease and Microbiome Program, Broad Institute of MIT and Harvard, Cambridge, MA, USA

The authors have addressed the comments in my initial review and the genome assemblies are now public. I have a few minor comments on this revised version. In Table 1, I would recommend removing the top row and instead in the table title referring to the kmer size and sequence coverage, or alternatively to merge the cells in the top row- as is, those cells appear to refer to the columns below and not the entire table. For Table 2- the Spades Hybrid has the same read stats as the other nanopore only assemblies but lists using both illumina and nanopore data. For Figure 4- was the 'before' data computed for the 2 Spades assemblies- seems odd that there were no BUSCO hits in those pre-pilon versions.

I have read this submission. I believe that I have an appropriate level of expertise to confirm that it is of an acceptable scientific standard.

# Peer review: Referee response for version 1

**DOI:** 10.5256/f1000research.12025.r23807

**Published:** 2017-07-27

**Authors:** Hayan Lee

**Affiliations:** 1 Department of Genetics, School of Medicine, Stanford University, California, CA, USA

Jansen *et al*. used Oxford Nanopore Technology with other short read sequencing technology, HiSeq 2500, to perform high-quality *de novo* genome assembly and classify yeast strain isolates, *Candida vartiovaarae* DDNA#1 from *Saccharomyces cerevisiae* S288C and *Cyberlindrena jadinii* CBS1600/NBRC 0988. They also exploited two versions of Nanopore flowcell chemistry and related software. Especially AT-rich mitochondria assembly using R7.3 and R9 comparison is very interesting. 

 Using similar short read data, N50 of DDNA#1 is 2.2kbp and that of S277C was 124Kbp. Probably authors want to perform repeat analysis for both strains to further study what makes such a performance gap. 

 For assembly approach two and three, authors used Canu to correct Nanopore reads with short reads. 

 So basically all three approaches adopted short reads for correction or assembly purpose. Since Canu can perform self-correction with only long reads, it would be very interesting to compare self-corrected Nanopore reads assembly contiguity vs. short reads corrected Nanopore reads assembly contiguity. 

 Authors used two error correction methods; Canu and PILON, It would be helpful to consistently compare the correction performance of two software. 

 Although *C. jadinii *stains are proposed to be the closest strain, given Figure 4, S288C looks much closer to DDNA#1. Probably authors want to take a close look at this. 

 All sequencing data should be available online for reproducibility.

I have read this submission. I believe that I have an appropriate level of expertise to confirm that it is of an acceptable scientific standard, however I have significant reservations, as outlined above.

# Response

**Published:** 2018-07-04

**Authors:** Michael Liem

**Affiliations:** Leiden University, The Netherlands

Using similar short read data, N50 of DDNA#1 is 2.2kbp and that of S277C was 124Kbp. Probably authors want to perform repeat analysis for both strains to further study what makes such a performance gap.

- **We hope to have shown that the performance gap can be overcome using long reads. Using long reads in either hybrid or with data exclusively from nanopore decreased fragmentation and increased contiguity. Suggesting genomic complexity caused initial difficulties during assembly of this strain.**

For assembly approach two and three, authors used Canu to correct Nanopore reads with short reads. So basically all three approaches adopted short reads for correction or assembly purpose. Since Canu can perform self-correction with only long reads, it would be very interesting to compare self-corrected Nanopore reads assembly contiguity vs. short reads corrected Nanopore reads assembly contiguity.

- **We have compared Canu (self-corrected) results to assemblies made with Miniasm, TULIP and Smartdenovo corrected with Racon. It appears the assembly strategy is a crucial difference to contiguity and fragmentation as opposed to self- or post-assembly correction.**

Authors used two error correction methods; Canu and PILON, It would be helpful to consistently compare the correction performance of two software.

- **The comparison between different assemblers and correction procedures should be more consistent now that we have separated the two task more prominently.**

Although *C. jadinii *stains are proposed to be the closest strain, given Figure 4, S288C looks much closer to DDNA#1. Probably authors want to take a close look at this.

- **Although alignment hits between C. jadinii and S288C are more targeted towards the diagonal in this figure the alignment length is very short and the number of alignment hits is significantly lower compared to the other two strains. This underlines the poor synteny conservation between C. jadinii and S288C as compared to CBS1600 and NBRC 0988. C. jadinii compared to these two strains show many more alignment hits hence these strains are taken to be more similar.**

All sequencing data should be available online for reproducibility.

- **Data has status in process, should be publicly accessible very soon**

# Peer review: Referee response for version 1

**DOI:** 10.5256/f1000research.12025.r24005

**Published:** 2017-07-17

**Authors:** Christina A. Cuomo

**Affiliations:** 1 Infectious Disease and Microbiome Program, Broad Institute of MIT and Harvard, Cambridge, MA, USA

This report by Jansen *et al* describes comparison of *de novo *assemblies generated using Illumina or Oxford Nanopore sequence for the yeast Candida varitovaarae.  The sequenced isolate was collected from a screen for new ethanologenic yeast species.  Genomic DNA was sequenced using both platforms and *de novo* assemblies compared for overall metrics and representation of the mitochondrial genome.  The final assembly was compared to those of other related yeast species to view conservation of synteny.

 Overall this is an interesting study in showing the advantage of utilizing long Oxford nanopore reads for assembly of a genome that was difficult to assemble using Illumina data. This description would be more compelling if the authors could address a few issues with the presentation of this data.

 1. In addition to genome size, the major factors that can influence the outcome of a *de novo *assembly are the repetitive sequence content, GC content, and level of heterozygosity.  The authors suggest that repetitive sequence could explain large number of contigs; this could be directly addressed by identifying repetitive sequences in the assembly and evaluating contig ends. However there is also the suggestion in the text of some level of heterozygosity, which could better account for the low contig N50 they report in the Illumina assemblies.  Whether or not the species is diploid and if so the level of heterozygosity is important to address in evaluating the performance of the two sequencing approaches and documenting the genomes for which long reads are most useful. This could be addressed for example using the Illumina data to identify heterozygous variants across the assembly.

 2. The authors use Pilon to correct the assembled contigs with Illumina data and note that this led to a minor increase in size of the assembly, suggesting there were some misassembled regions in the original Canu assembly. As the other genomes compared using Nucmer are distantly related, with many rearrangements, this could not be used to validate the Canu assembly.  It would be helpful if the authors could more fully describe the errors identified and fixed by Pilon. 

 3. Along the same lines, which statistics are for the final, best version of the assembly?  Table 1 compares different combinations of Oxford Chemistry, however the authors also describe an additional step of Pilon polishing. It would be useful to contrast metrics, including sequence coverage levels and GC content, to those from the 2 Spades assemblies, as well as note which assembly is the final version.

 4. In Figure 1, the top scale is too small to read. Plotting the GC as a separate track would be helpful to compare to the R7 coverage level.

 5. For the PFG in Figure 3, a longer run may help separate the bright high MW band into separate chromosomes.

 6. The data does not appear to be submitted to a public repository; both the raw sequence and the final best assembly should be submitted to NCBI or the ENA.

I have read this submission. I believe that I have an appropriate level of expertise to confirm that it is of an acceptable scientific standard, however I have significant reservations, as outlined above.

# Response

**Published:** 2018-07-04

**Authors:** Michael Liem

**Affiliations:** Leiden University, The Netherlands

1. In addition to genome size, the major factors that can influence the outcome of a *de novo *assembly are the repetitive sequence content, GC content, and level of heterozygosity. The authors suggest that repetitive sequence could explain large number of contigs; this could be directly addressed by identifying repetitive sequences in the assembly and evaluating contig ends. However there is also the suggestion in the text of some level of heterozygosity, which could better account for the low contig N50 they report in the Illumina assemblies. Whether or not the species is diploid and if so the level of heterozygosity is important to address in evaluating the performance of the two sequencing approaches and documenting the genomes for which long reads are most useful. This could be addressed for example using the Illumina data to identify heterozygous variants across the assembly.

- **The estimated genome size comparison between nanopore mediated assemblies and hybrid Spades assembly is a first indication of the polyploid genome of our strain. Together with the abundant double gene copy BUSCO gene identification analysis and Spades hybrid contigs alignment to TULIP contigs we hope to have shown the diploid characteristics of DDNA#1, at least to partial extend.**

 

2. The authors use Pilon to correct the assembled contigs with Illumina data and note that this led to a minor increase in size of the assembly, suggesting there were some misassembled regions in the original Canu assembly. As the other genomes compared using Nucmer are distantly related, with many rearrangements, this could not be used to validate the Canu assembly. It would be helpful if the authors could more fully describe the errors identified and fixed by Pilon.

- **Increased assembly length after PILON correction is mainly due to corrected homopolymer stretches that are often underrepresented due to sequencing complexities of low complexity regions. This explanation has been added to the manuscript under results and discussion – Illumina and MinION *de novo* genome assembly.**

 

3. Along the same lines, which statistics are for the final, best version of the assembly? Table 1 compares different combinations of Oxford Chemistry, however the authors also describe an additional step of Pilon polishing. It would be useful to contrast metrics, including sequence coverage levels and GC content, to those from the 2 Spades assemblies, as well as note which assembly is the final version.

- **The final assembly is now described under results and discussion – Illumina and MinION *de novo* genome assembly. We have added sequence data statistics such as coverage and total amount of data. And aimed to highlight the error correction effect using BUSCO gene identification analysis. **

 

4. In Figure 1, the top scale is too small to read. Plotting the GC as a separate track would be helpful to compare to the R7 coverage level.

- **GC-content is now added to this figure and numbers and text have been made more clear.**

 

5. For the PFG in Figure 3, a longer run may help separate the bright high MW band into separate chromosomes.

- **We have tried many different run conditions and failed to properly resolve the largest bands. This may be different on a different system but we do not have access to such a system.**

 

6. The data does not appear to be submitted to a public repository; both the raw sequence and the final best assembly should be submitted to NCBI or the ENA.

- **Data has status in process, should be publicly accessible very soon**

# Peer review: Referee response for version 1

**DOI:** 10.5256/f1000research.12025.r23808

**Published:** 2017-07-07

**Authors:** Jean-Marc Aury, Istace Benjamin

**Affiliations:** 1 Genoscope, Institut de biologie François-Jacob, Commissariat à l’Energie Atomique (CEA), Université Paris-Saclay, Evry, F-91057, France

We read the manuscript by Jansen *et al.* titled “ *De novo* whole-genome assembly of a wild type yeast isolate using Nanopore sequencing” with great interest. Authors describe their strategy to sequence and assemble a yeast strain using different methodologies: a short read strategy with Illumina reads alone and two hybrid approaches, the first one combining both short and long reads for the assembly and the second using long reads for the assembly and short reads for the correction of the consensus. In general, we think that this is a well put together study that reflects the current standard approaches for assembling genomes with both short and long reads. However, we have some questions/remarks that we would like the authors to answer.

1. It seems that the high level of polymorphism complicate the *de novo *assembly. If some regions are heterozygous, it should lead to a higher than expected assembly size. We think the authors should describe in more details the Illumina-only assembly especially the cumulative size (add a column in Table 1). As the error rate is low, with a high level of SNPs, both (Is the DDNA#1 isolate is a diploid yeast?) haplotypes should be segregated. On the contrary, the assembly length of the nanopore-only assemblies seems to be near the expected size (12Mb), does it mean that the error rate prevent to distinguish haplotypes? We think the authors should discuss in more details how haplotypes are resolved in their different assemblies.
2. The whole dataset (reads + final assembly) should be submitted in public repository to ensure full reproducibility.
3. Paragraph **Illumina and MinION *de novo* genome assembly, line 38. **Contigs were polished using the Pilon tool but line 7 of the same paragraph, authors indicate that the Spades assembly that was generated from Illumina reads alone was highly fragmented possibly due to a high level of SNPs in the DDNA#1 isolate. I think that to verify if the Pilon correction didn’t do more harm than good, authors could run the Busco tool ( http://busco.ezlab.org/) on the assemblies, or annotate genes, before and after correction to verify if it didn’t introduce errors in the consensus due to heterogeneous input reads.
4. Paragraph **Illumina and MinION de novo genome assembly, lines 14-15** it is said that the cumulative size of reads that was given as input to Canu was 2.05 Gb and that the corrected reads cumulative output size was equal to 389 Mb. I think that by default Canu only corrects 30X of the input read set (controlled by the corOutCoverage parameter) and since it is relatively close to 30-fold coverage of a yeast genome, I was wondering if authors leaved this parameter as default or if they moved up the limit and it could only correct around 30X of coverage. If this parameter was changed, I think it would be a good idea to indicate it.
5. Authors should add a table that contains standard metrics about the sequencing data (nanopore and illumina): number of reads, cumulative size, coverage, average read length…
6. Paragraph **Full genome comparison, lines 12-15** it is said that the Nucmer’s ouput was filtered with the delta-filter software; please add the parameters used to filter out alignments. Moreover, if the yeast genomes used for the comparison are highly variable the nucmer software is not the best suited; maybe lastz ( https://github.com/lastz/lastz) should better perform.
7. The smartdenovo assembler has been successfully applied to yeast genomes ( https://www.ncbi.nlm.nih.gov/pmc/articles/PMC5466710/), it would be interesting to compare their results with a smartdenovo assembly.

We have read this submission. We believe that we have an appropriate level of expertise to confirm that it is of an acceptable scientific standard, however we have significant reservations, as outlined above.

# Response

**Published:** 2018-07-04

**Authors:** Michael Liem

**Affiliations:** Leiden University, The Netherlands

1. It seems that the high level of polymorphism complicate the *de novo *assembly. If some regions are heterozygous, it should lead to a higher than expected assembly size. We think the authors should describe in more details the Illumina-only assembly especially the cumulative size (add a column in Table 1). As the error rate is low, with a high level of SNPs, both (Is the DDNA#1 isolate is a diploid yeast?) haplotypes should be segregated. On the contrary, the assembly length of the nanopore-only assemblies seems to be near the expected size (12Mb), does it mean that the error rate prevent to distinguish haplotypes? We think the authors should discuss in more details how haplotypes are resolved in their different assemblies.

- **Statistical information on the Illumina derived assembly is now added to Table 1. Indeed the majority of assemblies based exclusively on nanopore data are haploid genomes, this comes together with the notion that most of these assembler are designed to reconstruct bacterial genomes. However Canu should be able to differentiate diploid haplotypes, that is for high coverage datasets. It appears 17x coveragehigh quality long length read data is insufficient to resolve the (partial) diploid genome of DDNA#1.**

 

2. The whole dataset (reads + final assembly) should be submitted in public repository to ensure full

reproducibility.

- **These should be publicly available now**

 

3. Paragraph **Illumina and MinION** ***de novo*** **genome assembly, line 38. **Contigs were polished using the Pilon tool but line 7 of the same paragraph, authors indicate that the Spades assembly that was generated from Illumina reads alone was highly fragmented possibly due to a high level of SNPs in the DDNA#1 isolate. I think that to verify if the Pilon correction didn’t do more harm than good, authors could run the Busco tool (http://busco.ezlab.org/) on the assemblies, or annotate genes, before and after correction to verify if it didn’t introduce errors in the consensus due to heterogeneous input reads.

- **Thank you for your suggestion this is now incorporated into the manuscript under methods/ results and discussion – genome assembly assessment based on gene expectation using BUSCO**

 

4. Paragraph **Illumina and MinION de novo genome assembly, lines 14-15 **it is said that the cumulative size of reads that was given as input to Canu was 2.05 Gb and that the corrected reads cumulative output size was equal to 389 Mb. I think that by default Canu only corrects 30X of the input read set (controlled by the corOutCoverage parameter) and since it is relatively close to 30-fold coverage of a yeast genome, I was wondering if authors leaved this parameter as default or if they moved up the limit and it could only correct around 30X of coverage. If this parameter was changed, I think it would be a good idea to indicate it.

- **In our Canu version corOutCoverage is set to 40x coverage by default and has not been changed.**

 

5. Authors should add a table that contains standard metrics about the sequencing data (nanopore

and illumina): number of reads, cumulative size, coverage, average read length…

- **Table is now added.**

 

6. Paragraph **Full genome comparison, lines 12-15 **it is said that the Nucmer’s ouput was filtered with the delta-filter software; please add the parameters used to filter out alignments. Moreover, if the yeast genomes used for the comparison are highly variable the nucmer software is not the best suited; maybe lastz (https://github.com/lastz/lastz) should better perform.

- **Thank you for your suggestion, we have performed similar whole genome alignments with Lastz and mummer, however we didn’t observe a noticeable difference based on whole genome comparison alone. It appears the assembly algorithm and input data characteristics are the major factors that influenced the contiguity and fragmentation of our assemblies.**

 

7. The smartdenovo assembler has been successfully applied to yeast genomes (https://www.ncbi.nlm.nih.gov/pmc/articles/PMC5466710/), it would be interesting to compare their results with a smartdenovo assembly.

- **Thank you for your suggestion, Smartdenovo has now been added to the set of assemblers and results are denoted in our manuscript. Indeed Smartdenovo is an assembler that performs relatively well on the dataset of our yeast strain.**

# Peer review: Referee response for version 1

**DOI:** 10.5256/f1000research.12025.r23377

**Published:** 2017-06-27

**Authors:** Mile Šikić

**Affiliations:** 1 Faculty of Electrical Engineering and Computing, University of Zagreb, Zagreb, Croatia

The authors presented *de novo* whole-genome assembly of a wild type yeast isolate using nanopore sequencing. They tried three different approaches to assemble the genome: using Illumina reads only, using both Illumina and nanopore reads in a hybrid approach, and using the only nanopore reads for assembling and Illumina reads for polishing. The third approach resulted in the most contiguous assembly. In they work they use nanopore datasets made with R7.3, R9 and R9.4 chemistries.

 Although they used a correct procedure for genome assembly it would be interesting to compare their results with the following methods in the third approach:

- Using minimap+ miniasm assembler in combination with Racon consensus tool and PILON
- Using Canu + racon + PILON
- Try to polish nanopore assembly using Nanopolish

In addition, it would be valuable if they make their data publicly available to enable others to reproduce their results.

I have read this submission. I believe that I have an appropriate level of expertise to confirm that it is of an acceptable scientific standard, however I have significant reservations, as outlined above.

# Response

**Published:** 2018-07-04

**Authors:** Michael Liem

**Affiliations:** Leiden University, The Netherlands

Using minimap+ miniasm assembler in combination with Racon consensus tool and PILON

- **Thank you for your suggestion, this strategy is now included in our study.**

 

Using Canu + racon + PILON

- **Since Canu contains an integrated self-correction procedure prior to assembly we have not corrected the Canu contigs with Racon, however the combination Canu – PILON correction is part of our study, thank you.**

 

Try to polish nanopore assembly using Nanopolish

- **Thank you for your suggestion, however, since we have combined different data sets from different chemistries and different laboratories, at different times, including filtering of these data, it’s relatively complicated to polish such datasets with Nanopolish. To balance out the effort-result ratio we have performed a double iteration PILON correction which shows to be sufficient to identify the majority of genes stored in the Fungi 0db9 database used by BUSCO.**

 

In addition, it would be valuable if they make their data publicly available to enable others to reproduce their results.

- **Data has status in process, should be publicly accessible very soon**
